# Development and validation of a RNA binding protein-associated prognostic model for lung adenocarcinoma

**DOI:** 10.18632/aging.102828

**Published:** 2020-02-22

**Authors:** Wei Li, Li-Na Gao, Pei-Pei Song, Chong-Ge You

**Affiliations:** 1 Laboratory Medicine Center, Lanzhou University Second Hospital, Lanzhou 730030, China

**Keywords:** lung adenocarcinoma, RNA binding proteins, overall survival, prognostic model

## Abstract

RNA binding proteins (RBPs) dysregulation have been reported in various malignant tumors and associated with the occurrence and development of cancer. However, the role of RBPs in lung adenocarcinoma (LUAD) is poorly understood. We downloaded the RNA sequencing data of LUAD from the Cancer Genome Atlas (TCGA) database and determined the differently expressed RBPs between normal and cancer tissues. The study then systemically investigated the expression and prognostic value of these RBPs by a series of bioinformatics analysis. A total of 223 differently expressed RBPs were identified, including 101 up-regulated and 122 down-regulated RBPs. Eight RBPs (*IGF2BP1*, *IFIT1B*, *PABPC1*, *TLR8*, *GAPDH*, *PIWIL4*, *RNPC3*, and *ZC3H12C*) were identified as prognosis related hub gene and used to construct a prognostic model. Further analysis indicated that the patients in the high-risk subgroup had poor overall survival(OS) compared to those in low-risk subgroup based on the model. The area under the curve of the time-dependent receiver operator characteristic curve of the prognostic model are 0.775 in TCGA cohort and 0.814 in GSE31210 cohort, confirming a good prognostic model. We also established a nomogram based on eight RBPs mRNA and internal validation in the TCGA cohort, which displayed a favorable discriminating ability for lung adenocarcinoma.

## INTRODUCTION

Lung cancer is a very harmful disease that remains a top cause of cancer-related deaths worldwide. It is estimated at 220,000 newly diagnosed lung cancer cases and more than 140,000 deaths in the USA in 2019 [1]. Non-small cell lung cancer (NSCLC) is the most common type of lung cancer, including lung squamous cell carcinoma and lung adenocarcinoma (LUAD). LUAD is a major component of lung cancer, accounting for approximately 40% of all lung cancer patients [2]. Even though there has been great progress in the diagnostic and treatment methods over the past few decades, the average 5-year relative survival rate of lung cancer is only 18% [3]. At present, the diagnosis of lung cancer primarily depends on histopathological examination, cancer molecular biomarkers, imaging evaluations, and it is difficult to achieve early detection of lung tumor [4, 5]. This may be the most significant cause of high mortality in lung cancer patients. Therefore, further understanding the molecular mechanism of lung cancer to develop effective methods for early screening and diagnosis are critical to improve therapeutic effect and quality of life of patients.

RNA binding proteins (RBPs) are a class of proteins that interact with a variety of types of RNAs involve in rRNAs, ncRNAs, snRNAs, miRNAs, mRNAs, tRNAs, and snoRNAs. To date, more than 1,500 RBP genes have been identified by genome-wide screening in human genome [6]. These RBPs play important roles in maintaining the physiological balance of cells, especially during the development process and stress responses [7]. RBPs can bind to their target RNAs in a structure or sequence- dependent mode to form ribonucleoprotein complexes that regulate mRNA stability, RNA processing, splicing, localization, export, and translation at the post-transcriptional level [7]. Considering the importance of post-transcriptional regulation in life processes, it is thus not surprising that aberrantly deregulated RBPs are closely related to the occurrence and progression of numerous human diseases. Mutations in RNA-binding proteins localized in the central nervous system lead to aberrant protein aggregation, which promote the progression of various neurodegenerative diseases [8, 9]. Previous studies have indicated that RBPs such as SRSF1, Quaking, Muscleblind and HuR, as pivotal moderators to regulate the occurrence and progression of cardiovascular diseases by mediating a wide range of post-transcriptional events [10]. Even though RBPs are known to be involved in the initiation and development of various diseases, the roles of RBPs in tumor development is still rare.

In the past decades, many reports have revealed that RBPs were abnormally expressed in tumors, which affected the translation of mRNA into protein, and were involved in carcinogenesis [11–13]. Among them, only a few RBPs have been investigated in depth and found to play critical roles in human cancers. For example, HuR by regulating mRNA stability to promote proliferation and metastasis of gastric cancer [14]; AGO2 facilitates tumor progression via elevating oncogenic miR-19b biogenesis [15]; QKI-5 inhibit cancer-associated alternative splicing to regulate cell proliferation in lung cancer [16]; ESRP1 promotes the transformation of ovarian cancer cells from mesenchymal to epithelial phenotype [17]. A systematic functional study of RBPs will help us fully understand their roles in tumors. Therefore, we downloaded LUAD RNA-sequencing and clinicopathological data from the cancer genome atlas (TCGA) database. Subsequently, we identified aberrantly expressed RBPs between cancerous and normal samples by high-throughput bioinformatic analysis, and systematically explored their potential functions and molecular mechanisms. Our study determined a number of LUAD-related RBPs that promote our understanding of the molecular mechanisms underlying lung cancer progression. These RBPs might provide potential biomarkers for diagnosis and prognosis.

## RESULTS

### Identification of differently expressed RBPs in LUAD patients

**Figure 1:**
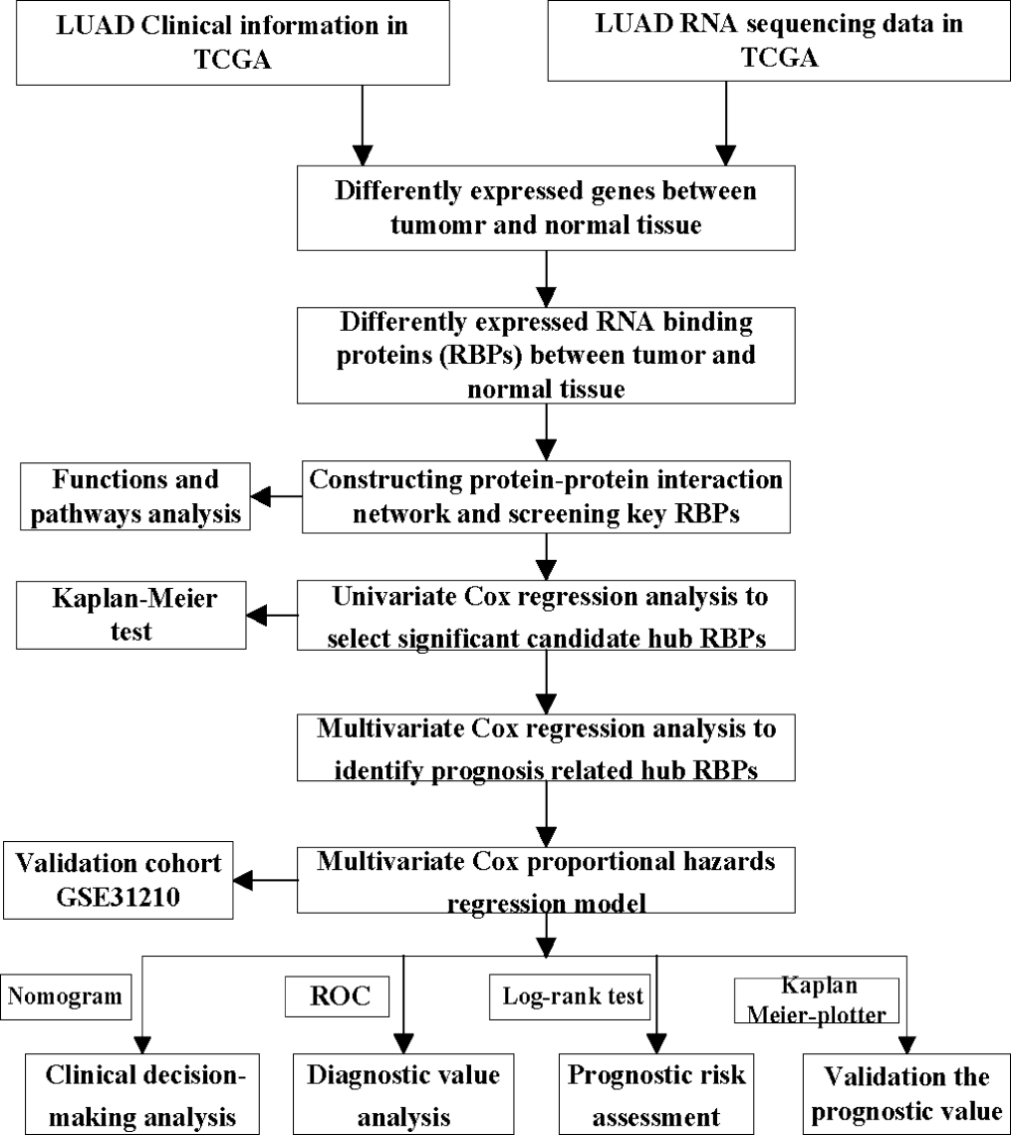
**Whole procedures for analyzing RBPs in lung adenocarcinoma.**

**Figure 2:**
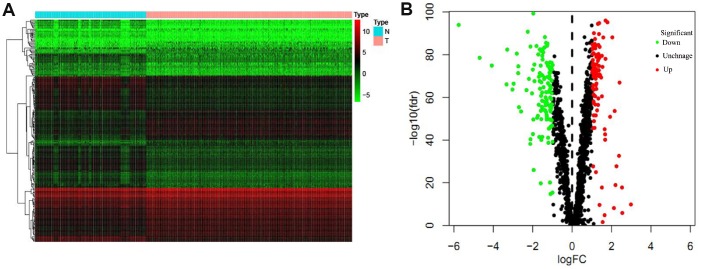
**The differentially expressed RBPs in lung adenocarcinoma.** (**A**) Heat map; (**B**) Volcano plot.

In this study, we conducted a systematic analysis of key roles and prognostic values of RBPs in LUAD by several advanced computational methods. The study design was illustrated in Figure 1. The databases of lung adenocarcinoma were downloaded from TCGA contained 524 tumor samples and 59 normal lung tissue samples. The R software packages were applied to handle the data and discover the differently expressed RBPs. A total of 1542 RBPs [6] were included in the analysis, and 223 RBPs met the screening standard of this study *(P*<0.05, |log2FC)| >1.0), which consist of 101 upregulated and 122 downregulated RBPs. The expression distribution of these differently expressed RBPs was displayed in Figure 2.

### GO and KEGG pathway enrichment analysis of the differently expressed RBPs

To investigate the function and mechanisms of the identified RBPs, we divided these differently expressed RBPs into two groups: up-regulated or down-regulated expression. Then we uploaded these differently expressed RBPs to the online tool WebGestalt for functional enrichment analysis. The results indicated that downregulated differently expressed RBPs were significantly enriched in the biological process related to negative regulation of translation, RNA phosphodiester bond hydrolysis, regulation of mRNA metabolic process, regulation of translation, and mRNA processing (Table 1). The upregulated differently expressed RBPs were significantly enriched in organonitrogen compound biosynthetic process, cellular amide metabolic process, RNA processing, peptide metabolic process, and amide biosynthetic process (Table 1). In terms of molecular function, the decreased differently expressed RBPs were notably enriched in RNA binding, mRNA binding, ribonuclease activity, double-stranded RNA binding and mRNA 3'-UTR binding (Table 1), while the upregulated differently expressed RBPs were significantly enriched in RNA binding, structural constituent of ribosome, mRNA binding, structural molecule activity, and catalytic activity, acting on RNA (Table 1). Through the cellular component (CC) analysis, we found that the decreased differently expressed RBPs were enriched in micro-ribonucleoprotein complex, ELL-EAF complex, RISC complex, micro-ribonucleoprotein complex, and ribonucleoprotein complex, and upregulated differently expressed RBPs were mainly enriched in ribosome, ribosomal subunit, ribonucleoprotein complex, large ribosomal subunit, and cytosolic ribosome (Table 1). Moreover, we found that downregulated differently expressed RBPs were mainly enriched in mRNA surveillance pathway, RNA degradation, and Ribosome biogenesis in eukaryotes, while upregulated RBPs were significantly enriched for Ribosome, Spliceosome, and RNA degradation (Table 1).

**Table 1:** KEGG pathway and GO enrichment analysis of aberrantly expressed RBPs.

	**GO term**	***P* value**	**FDR**
**Down-regulated RBPs**			
Biological processes	negative regulation of translation	4.27E-14	3.89E-11
	RNA phosphodiester bond hydrolysis	2.00E-14	2.25E-11
	regulation of mRNA metabolic process	3.33E-15	4.33E-12
	regulation of translation	1.11E-15	1.68E-12
	mRNA processing	0	0
Cellular component	micro-ribonucleoprotein complex	6.81E-10	1.60E-7
	ELL-EAF complex	0.000002	0.000195
	RISC complex	1.15E-7	0.000017
	micro-ribonucleoprotein complex	6.81E-10	1.60E-7
	ribonucleoprotein complex	0	0
Molecular function	RNA binding	0	0
	mRNA binding	0	0
	ribonuclease activity	1.16E-11	5.00E-9
	double-stranded RNA binding	2.24E-12	1.40E-9
	mRNA 3'-UTR binding	3.73E-8	8.76 E-6
KEGG pathway	mRNA surveillance pathway	1.25E-7	4.07 E-5
	RNA degradation	0.000025	0.004063
	Ribosome biogenesis in eukaryotes	0.000457	0.049713
**Up-regulated RBPs**			
Biological processes	organonitrogen compound biosynthetic process	0	0
	cellular amide metabolic process	0	0
	RNA processing	0	0
	peptide metabolic process	0	0
	amide biosynthetic process	0	0
Cellular component	ribonucleoprotein complex	0	0
	ribosome	0	0
	ribosomal subunit	0	0
	large ribosomal subunit	0	0
	cytosolic ribosome	7.23E-15	1.70E-12
Molecular function	RNA binding	0	0
	structural constituent of ribosome	0	0
	mRNA binding	6.39E-11	3.93E-8
	structural molecule activity	8.37E-11	3.93E-8
	catalytic activity, acting on RNA	1.42E-9	5.31E-7
KEGG pathway	Ribosome	0	0
	Spliceosome	1.03E-9	1.67E-7
	RNA degradation	0.000005	0.000503

### Protein-protein interaction (PPI) network construction and key modules selecting

**Figure 3:**
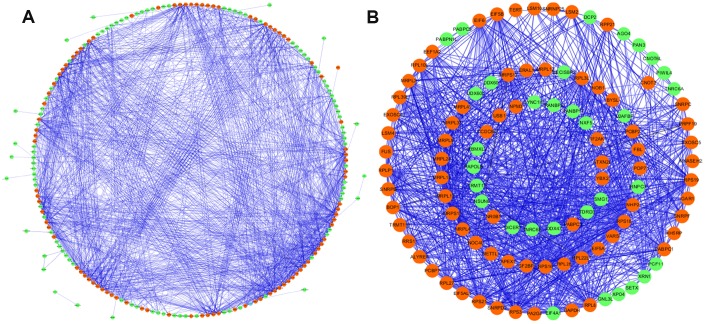
**Protein-protein interaction network and modules analysis.** (**A**) Protein-protein interaction network of differentially expressed RBPs; (**B**) critical module from PPI network. Green circles: down-regulation with a fold change of more than 2; red circles: up-regulation with fold change of more than 2.

To further investigated the roles of differently expressed RNA binding proteins in LUAD, we created the PPI network using Cytoscape software which incorporated 197 nodes and 1484 edges based on the data from STRING database (Figure 3A). The co-expression network was processed via using the MODE tool to identify possible key modules and the first important modules acquired, which consist of 107 nodes and 1088 edges (Figure 3B). The RBPs in the key module 1 were greatly abounded in mRNA surveillance pathway, RNA transport, RNA degradation, RNA processing, ribosome biogenesis in eukaryotes, ribonucleoprotein complex biogenesis, RNA binding, peptide metabolic process, amide biosynthetic process, and translation.

### Prognosis-related RBPs selecting

**Figure 4:**
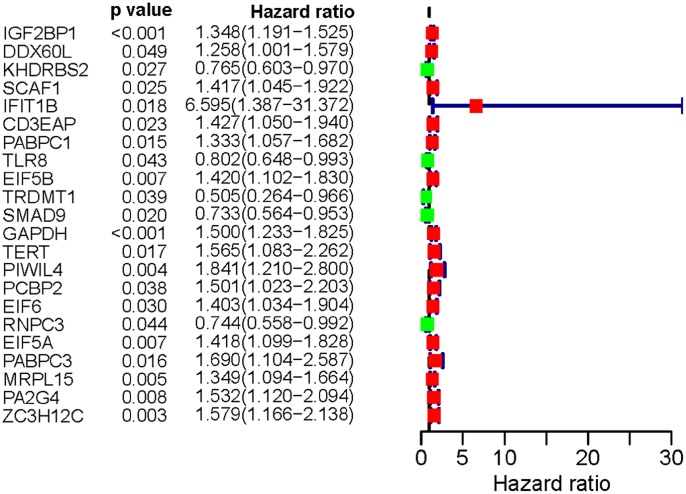
**Univariate Cox regression analysis for identification of hub RBPs in the training dataset.**

**Figure 5:**
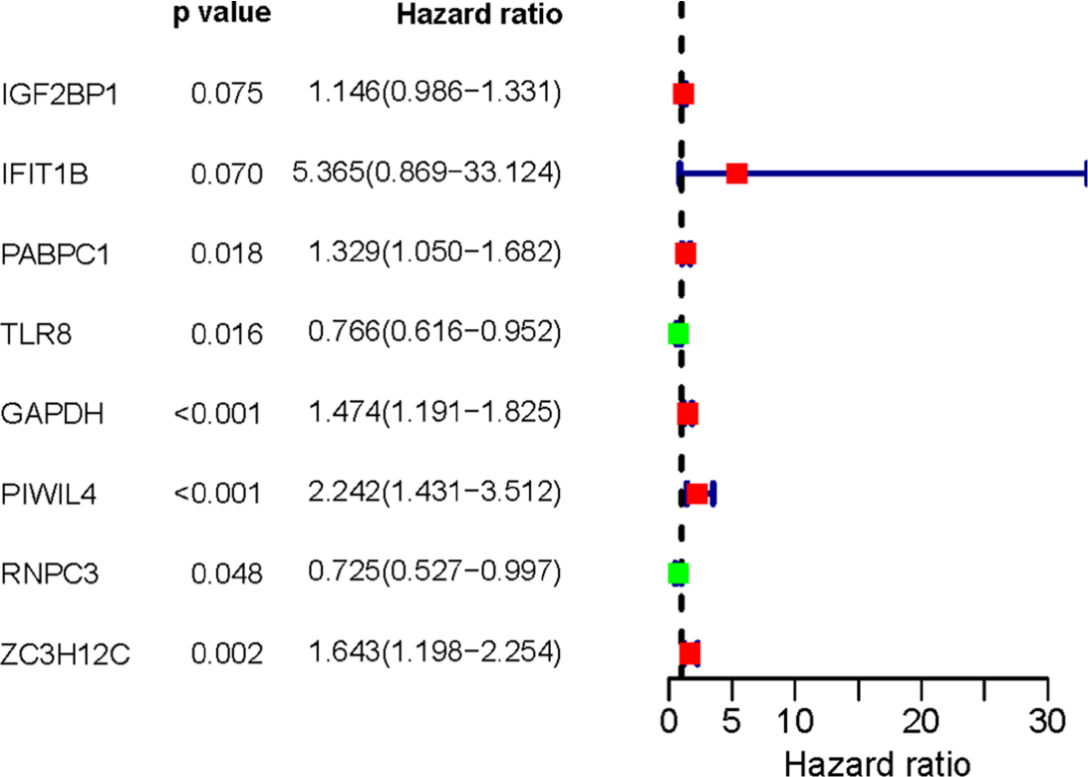
**Multivariate Cox regression analysis to identify prognosis related hub RBPs.**

A total of 197 key differently expressed RBPs were identified from the PPI network. To investigate the prognostic significance of these RBPs, we performed a univariate Cox regression analysis and obtained 22 prognostic-associated candidate hub RBPs (Figure 4). Subsequently, these 22 prognostic-associated candidate hub RBPs were analyzed by multiple stepwise Cox regression to investigate their impact on patient survival time and clinical outcomes, eight hub RBPs were found to be independent predictors in LUAD patients (Figure 5, Table 2).

**Table 2:** Eight prognosis-associated hub RBPs identified by multivariate Cox regression analysis.

**RBP name**	**coef**	**HR**	**Lower 95% CI**	**Upper 95% CI**	**P-value**
IGF2BP1	0.1362	1.1459	0.9862	1.3314	0.0751
IFIT1B	1.6799	5.3652	0.8690	33.1242	0.0704
PABPC1	0.2843	1.3288	1.0495	1.6824	0.0181
TLR8	-0.2663	0.7662	0.6163	0.9524	0.0164
GAPDH	0.3882	1.4743	1.1911	1.8248	0.0003
PIWIL4	0.8073	2.2419	1.4312	3.5117	0.0004
RNPC3	-0.3219	0.7247	0.5265	0.9974	0.0481
ZC3H12C	0.4965	1.6430	1.1976	2.2539	0.0021

### Prognosis-related genetic risk score model construction and analysis

The eight hub RBPs identified from the multiple stepwise Cox regression analysis were used to construct the predictive model. The risk score of each patient was calculated according to the following formula:

$$\begin{array}{l} Risk\;score=(0.1362*\text{ExpIGF}2\text{BP}1)+(1.6799*\text{ExpIFIT}1\text{B})\\ \;\;\;\;\;\;+(0.2843*\text{ExpPABPC}1)+(-0.2663*\text{ExpTLR}8)\\ \;\;\;\;\;\;+(0.3882*\text{ExpGAPDH}1)+0.8073*\text{ExpPIWIL}4\\ \;\;\;\;\;\;+(-0.3219*\text{ExpRNPC}3)+(-0.4965*\text{ExpZC}3\text{H}12\text{C}). \end{array}$$

**Figure 6:**
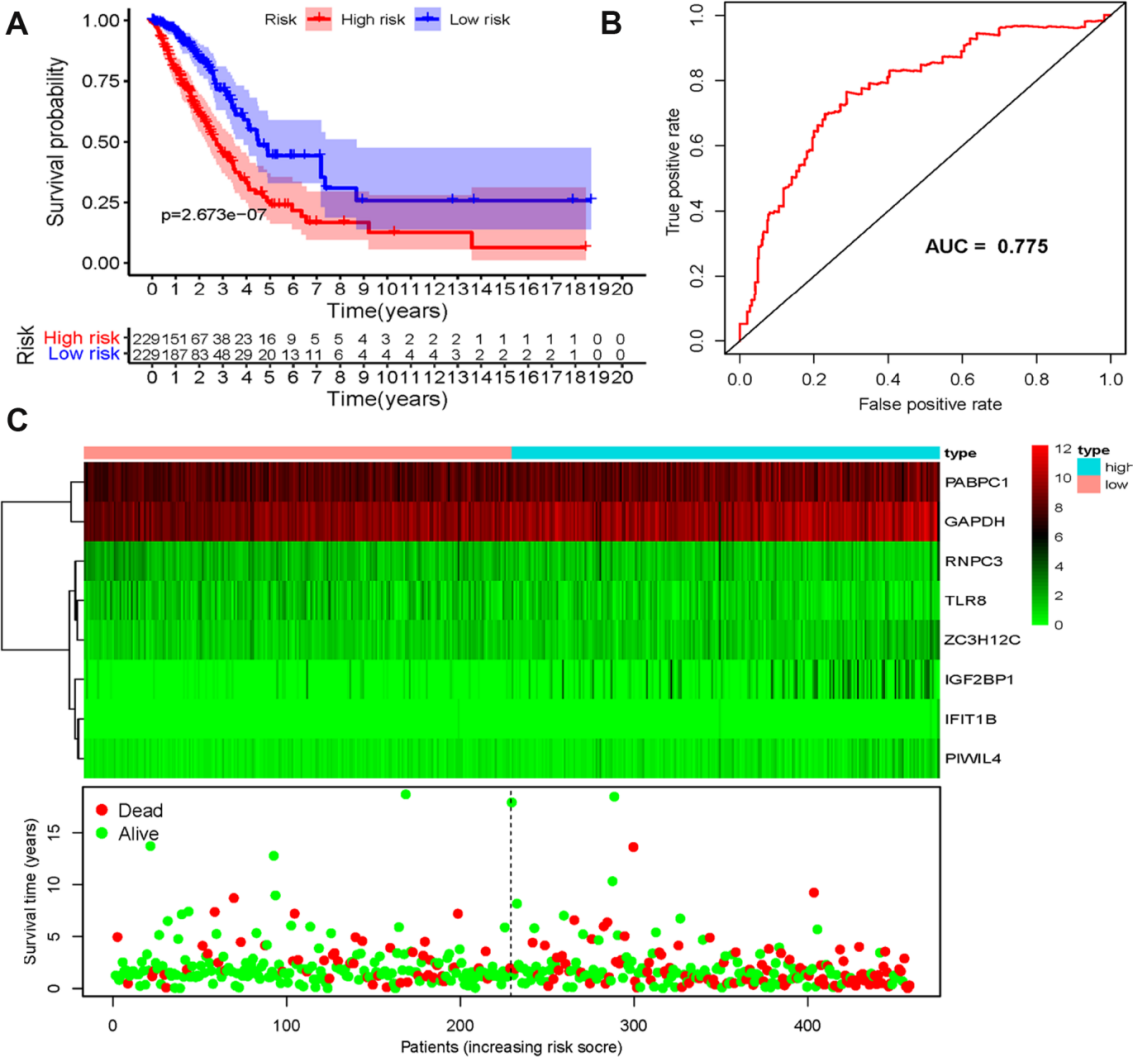
**Risk score analysis of eight-genes prognostic model in the TCGA cohort**. (**A**) Survival curve for low- and high-risk subgroups; (**B**) ROC curves for forecasting OS based on risk score; (**C**) Expression heat map, risk score distribution, and survival status.

**Figure 7:**
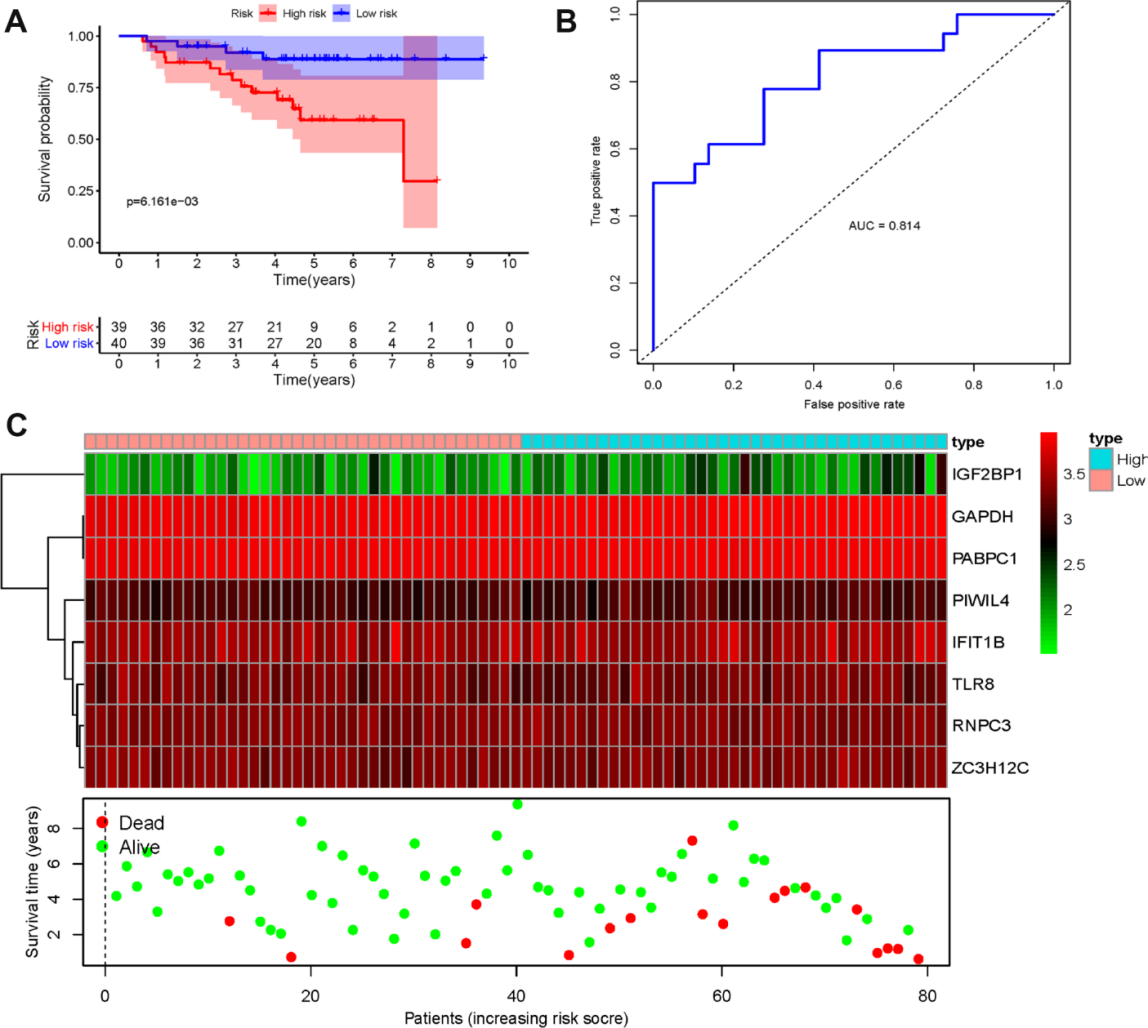
**Risk score analysis of eight-genes prognostic model in the GSE31210 cohort.** (**A**) Survival curve for low- and high-risk subgroups; (**B**) ROC curves for forecasting OS based on risk score; (**C**) Expression heat map, risk score distribution, and survival status.

We then conducted a survival analysis to assess the predictive ability. A total of 458 LUAD patients were divided into low-risk and high-risk subgroups according to the median risk score. The results indicated that the patients in the high-risk subgroup were with poor OS compared to those in the low-risk subgroup (Figure 6A). To further evaluate the prognostic ability of the eight-RBPs biomarker, a time-dependent ROC analysis was executed. We found that the area under the ROC curve (AUC) of this RBPs risk score model was 0.775 (Figure 6B), which indicated that it has moderate diagnostic performance. The expression heat map, survival status of patients, and risk score of the signature consisting of eight RBPs in the low- and high-risk subgroups are displayed in Figure 6C. In addition, we evaluated whether the eight-RBPs predictive model with similar prognostic value in other LUAD patient cohorts, the same formula was used to the GSE31210 datasets. We found that patients with high-risk score also have a poorer OS than those with low-risk score in the GSE31210 cohorts (Figure 7A–7C). These results suggested that the prognostic model has better sensitivity and specificity.

### Construction of a nomogram based on the eight hub RBPs

**Figure 8:**
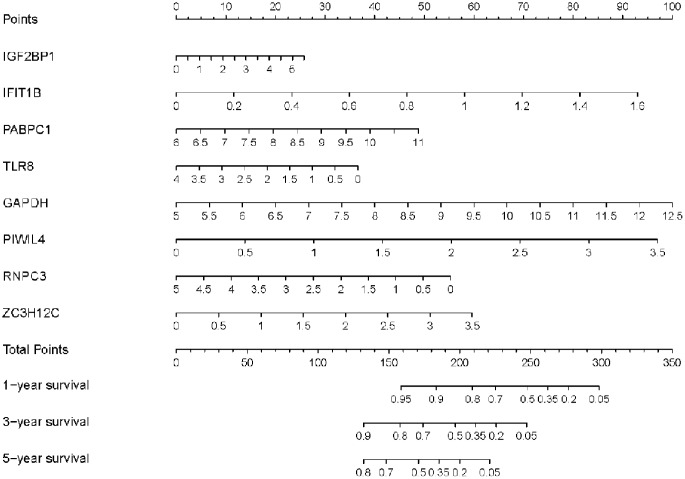
**Nomogram for predicting 1-, 3-, and 5-year OS of LUAD patients in the TCGA cohort.**

In order to develop a quantitative method for LUAD prognosis, we integrated the eight RBPs signature to establish a nomogram (Figure 8). Based on the multivariate Cox analysis, points were assigned to individual variables by using the point scale in the nomogram. We draw a horizontal line to determine the point of each variable and calculate the total points for each patient by summing the points of all variables, and normalize it to a distribution of 0 to 100. We can calculate the estimated survival rates for LUAD patients at 1, 3, and 5 years by drafting a vertical line between the total point axis and each prognosis axis, which might help relevant practitioners to develop clinical decision-making for LUAD patients. Besides, we assessed the prognostic significance of different clinical characteristics in LUAD patients from TCGA by performing COX regression analysis. The results showed that tumor stage, primary tumor site, regional lymph node involvement and risk score were correlated with OS of LUSC patients (*P*<0.01) (Table 3). However, we only found that age, tumor stage, and risk score were independent prognostic factors correlated with OS through multiple regression analysis (*P*<0.01) (Table 3).

**Table 3:** The prognostic value of different clinical parameters.

	**Univariate analysis**				**Multivariate analysis**		
	**HR**	**95% CI**	***P*-value**		**HR**	**95%CI**	***P*-value**
Age	1.02	0.99-1.03	0.154		1.03	1.01-1.05	0.002
Gender	1.02	0.70-1.49	0.909		0.88	0.59-1.31	0.536
Smoking	0.98	0.82-1.17	0.814		1.04	0.87-1.24	0.661
Stage	1.60	1.35-1.89	<0.001		1.50	1.20-1.88	<0.001
T	1.48	1.17-1.88	<0.001		1.08	0.83-1.41	0.551
N	1.44	1.23-1.69	<0.001		1.16	0.94-1.43	0.181
M	1.05	0.85-1.30	0.623		1.11	0.88-1.38	0.375
Risk score	1.21	1.15-1.27	<0.001		1.26	1.19-1.34	<0.001

### Validation the prognostic value and expression of hub RBPs

**Figure 9:**
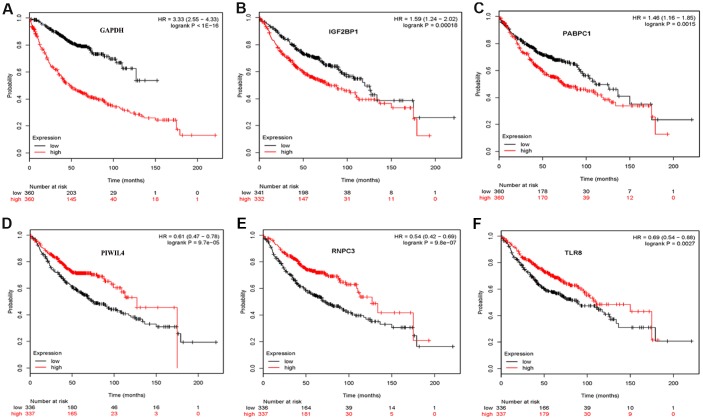
**Validation the prognostic value of hub RBPs in LUAD by Kaplan Meier-plotter.**

**Figure 10:**
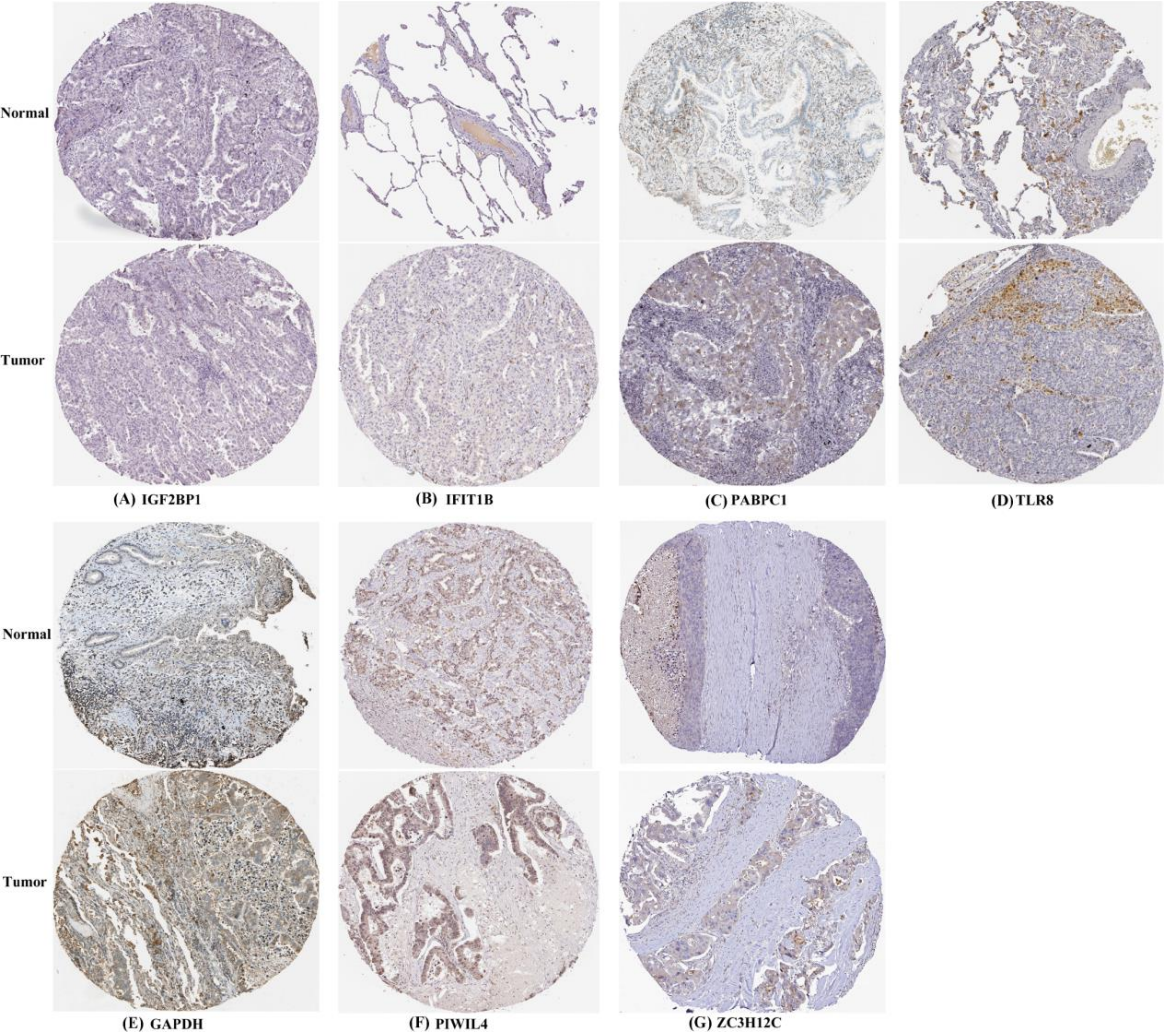
**Verification of hub RBPs expression in LUAD and normal lung tissue using the HPA database.** (**A**): IGF2BP1, (**B**): IFIT1B, (**C**): PABPC1, (**D**): TLR8, (**E**): GAPDH, (**F**): PIWIL4, (**G**): ZC3H12C.

To further explore the prognostic value of eight hub RBPs in LUAD, the Kaplan Meier-plotter was used to determine the relationship between hub RBPs and OS. A total of six of the eight hub RBPs (GAPDH, IGF2BP1, PABPC1, PIWIL4, RNPC3, and TLR8) were identified by Kaplan Meier-plotter server. The results of log-rank test demonstrated that the six RBPs were associated with the OS in LUAD patients (Figure 9). To further determine the expression of these hub RBPs in LUAD, we used immunohistochemistry results from the Human Protein Atlas database to show that IGF2BP1, PABPC1, and GAPDH were significantly increased in lung cancer compared with normal lung tissue (Figure 10). However, the antibody staining level of TLR8, PIWIL4, and ZC3H12C were relatively reduced in lung cancer tissue. Besides, the protein expression of IFIT1B was not significantly different between tumor and normal lung tissue (Figure 10).

## DISCUSSION

RBPs dysregulation has been reported in various malignant tumors [11, 18]. However, only a small part of RBPs have been studied in depth and partially confirmed that they contributed to occurrence and development of cancers [19]. In present study, we identified 223 differently expressed RBPs between tumor and normal tissue based on LUAD data from TCGA. We systematically analyzed relevant biological pathways, constructed co-expression network and PPI network of these RBPs. Moreover, we also performed univariate Cox regression analysis, survival analyses, multiple stepwise Cox regression analysis, and ROC analyses of hub RBPs to further explore their biological functions and clinical significance. We constructed a risk model to predict LUAD prognosis based on eight prognostic-associated hub RBP genes. These findings may contribute to develop novel biomarkers for the diagnosis and prognosis of patients with LUAD.

The function pathway enrichment analysis displayed that the differently expressed RBPs were greatly enriched in regulation of translation, RNA phosphodiester bond hydrolysis, regulation of mRNA metabolic process, RNA processing, organonitrogen compound biosynthetic process, cellular amide metabolic process, peptide metabolic process, amide biosynthetic process, RNA binding, ribonuclease activity, double-stranded RNA binding and mRNA 3'-UTR binding. Previous studies have proved that regulation of translation, RNA processing, and RNA metabolism are related to the occurrence and development of a variety of human diseases [20–22]. Post-transcriptional regulation of RNA stability is an important procedure in gene expression processing. RBPs can interact with RNA to form ribonucleoprotein complexes, thereby increasing the stability of target mRNAs and promoting gene expression, which play key roles in the progression of various diseases. Oncogenic RBP SRSF1 promotes lung cancer cell proliferation and development by enhancing the mRNA stability of DNA ligase 1 [23]. RBP SART3 binds pre-miR-34a with high specificity, and increased miR-34a levels to facilitate G1 cell cycle arrest in NSCLC cells [24]. Besides, ribonucleoprotein granule is a key region that executes protein biosynthesis. The alteration of ribonucleoprotein influences the translation processing and related to tumor progression [25]. The KEGG pathways analysis showed that the aberrantly expressed RBPs regulate the tumorigenesis and progression of lung carcinoma by affecting mRNA surveillance pathway, RNA degradation, ribosome biogenesis, and RNA degradation.

Moreover, we created a protein-protein interaction network of these differently expressed RBPs and got a key module including 107 key RBPs. Among these key RBPs, many of them have been shown to play an important role in the development and progression of tumors. EIF6, a eukaryotic translation initiation factor, affects the maturation of 60S ribosomal subunits, is upregulated in LUAD and negatively associated with patient prognosis [26, 27]. NOB1 is an important accessory factor in ribosome assembly, and upregulation of NOB1 expression can promote NSCLC cell growth [28]. Another study showed that NSCLC patients with high expression of NOB1 had a poor overall survival and progression-free survival [29]. Although the connection between the most of differently expressed RBPs and lung carcinoma remains unclear, some RBPs have been reported to be associated with other tumors. BOP1 as Wnt/β-catenin target gene involved in induced migration, EMT, and metastasis of colorectal carcinoma [30]. GNL3 can promote colon carcinoma cell proliferation, invasion and migration by activating the Wnt/β-catenin signaling pathway [31]. BYSL is upregulated in hepatocellular carcinoma, and as a crucial oncogene contributes to tumor cell growth both in vitro and in vivo [32]. DICER 1 as a ribonuclease, involving in the formation of mature microRNAs in the cytoplasm of all cancer cells. Many studies have shown that DICER 1 is dysregulation in multiple tumors, which is part of the pathological molecular mechanism that leads to the progression of this malignant tumor [33–35]. The module analysis of the PPI network showed that LUAD is related to mRNA surveillance pathway, RNA processing, RNA binding, ribosome biogenesis in eukaryotes, ribonucleoprotein complex biogenesis, peptide metabolic process, amide biosynthetic process, and translation.

Besides, the hub RBPs were selected based on univariate Cox regression analysis, survival analyses, and multiple Cox regression analysis. A total of eight RBPs were identified as prognosis related hub RBPs, including IGF2BP1, IFIT1B, PABPC1, TLR8, GAPDH, PIWIL4, RNPC3, and ZC3H12C. Previous studies have reported that the expression IGF2BP1 [36], TLR8 [37], PIWIL4 [38], and GAPDH [39] were associated with tumorigenesis and progression of lung cancer patients, which consistent with our results. Next, we produced a risk model to predict LUAD prognosis by multiple stepwise Cox regression analysis on the basis of the eight hub RBPs coding gene, trained using the TCGA cohort. The ROC curve analysis revealed that these eight genes signature with the better diagnostic capability to select out the LUAD patients with poor prognosis. However, the molecular mechanism of these eight RBPs contributes to lung carcinogenesis still poorly understood, and further exploration of potential mechanisms may be valuable. Subsequently, a nomogram was built to help us predict 1, 3, and 5 years OS more intuitively. We also used the Kaplan Meier-plotter to detect the prognostic value of the eight RBPs coding gene, the results were basically consistent with the prognostic analysis results of TCGA cohort. These results suggested that the prognostic model of eight-genes signature has a certain value in adjusting treatment plans of lung cancer patients.

Overall, our prognostic model is based on eight RBPs coding genes, which significantly reduces the cost of sequencing and is more conducive to clinical application. Besides, the eight genes predictive model has better performance for survival prediction in patients with LUAD. Moreover, the RBPs-associated gene signature displayed vital biological function, suggesting that they can potentially be used for clinical assistant treatment, which was not necessarily always the case in previous studies. Nonetheless, there are several limitations in this study. Firstly, our prognostic model was only based on the data from TCGA database, which is not validated in clinical patient cohort and other databases. Secondly, our study was designed on the basis of a retrospective analysis and prospective research should be performed to verify the outcomes. Thirdly, the datasets did not provide some clinical information, which may decrease the statistical validity and reliability of multivariate stepwise Cox regression analysis.

In summary, we systemically explored the expression and prognostic value of differently expressed RBPs by a series of bioinformatics analyses in LUAD. These RBPs may involve in tumorigenesis, progression, invasion and metastasis of LUAD. The prognostic model of eight RBPs coding gene was constructed, and which might serve as an independent prognostic factor for LUAD. As far as we know, this is the first report of developing a RBPs-associated prognostic model for LUAD. Our results would greatly contribute to show the pathogenesis of LUAD and to develop new treatment targets and prognostic molecular markers.

## MATERIALS AND METHODS

### Data processing

We downloaded the RNA-sequencing dataset of 59 normal lung tissue samples and 524 LUAD samples with corresponding clinical data from The Cancer Genome Atlas database (TCGA, https://portal.gdc.cancer.gov/). To identify the differently expressed genes between normal lung and LUAD tissue, we used the negative binomial distribution method. The Limma package (http://www.bioconductor.org/packages/release/bioc/html/limma.html) was applied to perform the analysis. The Limma package was based on the negative binomial distribution, it fits a generalized linear model for each gene and uses empirical Bayes shrinkage for dispersion and fold-change estimation. All raw data was preprocessed by Limma package and excluded genes with an average count value less than 1. In addition, we also used Limma package to identify the differently expressed RBPs in view of |log2 fold change (FC)|≥1 and false discovery rate (FDR)<0.05.

### KEGG pathway and GO enrichment analysis

The biological functions of these differently expressed RBPs were comprehensively detected by GO enrichment and kyoto encyclopedia of genes and genomes (KEGG) pathway analysis. The GO analysis terms including cellular component (CC), molecular function (MF), and biological process (BP). All enrichment analyses were carried out by utilizing online WEB-based Gene Set Analysis Toolkit (WebGestalt, http://www.webgestalt.org/) [40]. Both *P* and FDR values were less than 0.05 as statistically significant.

### PPI network construction and module screening

The differently expressed RBPs were submitted to the STRING database (http://www.string-db.org/) [41] to identify protein-protein interaction information. The Cytoscape 3.7.0 software was used to further construct the PPI network and visualized. The important modules and genes were elected in PPI network by using Molecular Complex Detection (MCODE) plug-in with both MCODE score and node counts number more than 5 [42]. All *P*≤ 0.05 were considered as significant difference.

### Prognostic model construction

Univariate Cox regression analysis was performed on all key RBPs in the key modules of the training dataset using survival R package. A log-rank test was executed to screen the significant candidate genes further. Subsequently, based on the above preliminary screened significant candidate genes, we constructed a multivariate Cox proportional hazards regression model and calculated a risk score to assess patient prognosis outcomes. The risk score formula for each sample was as follows:

Risk score=β1∗Exp1+β2∗Exp2+βi∗Expi,

where β represents the coefficient value, and Exp represented the gene expression level. According to the median risk score survival analysis, LUAD patients were divided into low-risk and high-risk groups. A log-rank test compared the difference of OS between the two subgroups. Additionally, a receiver operating characteristic (ROC) curve analysis was implemented by using the SurvivalROC package to evaluate the prognostic capability of the above model [43]. Besides, 79 LUAD patient samples with reliable prognostic information from the GSE31210 dataset (https://www.ncbi.nlm.nih.gov/ geo/query/acc.cgi?acc=GSE31210) were used as a validation cohort to confirm the predictive capability of this prognostic model. Finally, the nomogram with calibration plots was conducted using rms R package to forecast the likelihood of OS. *P*<0.05 was considered to be a significant difference.

### Verification of express level and prognostic significance

The Human Protein Atlas (HPA) online database (http://www.proteinatlas.org/) was used to detect the expression of eight hub RBPs at a translational level [44]. The prognostic value of the eight RBPs in LUAD was verified by using the Kaplan Meier plotter (https://kmplot.com/analysis/) online tool [45].
