# Correction for: Development and validation of a RNA binding protein-associated prognostic model for lung adenocarcinoma

**DOI:** 10.18632/aging.202826

**Published:** 2021-03-14

**Authors:** Wei Li, Li-Na Gao, Pei-Pei Song, Chong-Ge You

**Affiliations:** 1 Laboratory Medicine Center, Lanzhou University Second Hospital, Lanzhou, 730030, China

**Keywords:** correction

Original article: Aging. 2020; 12:3558–3573.  . https://doi.org/10.18632/aging.102828

**This article has been corrected: **Due to errors in figure preparation, some of the images in Figure 10 are incorrect. Specifically, the images for row 1 (normal), panels 1 and 3; and row 3 (normal), panels 1, 2 and 3, contain the wrong images. The corrected Figure 10, produced using the original data, is shown below. The authors declare that these corrections do not change the results or conclusions of this paper.

**Figure 10:**
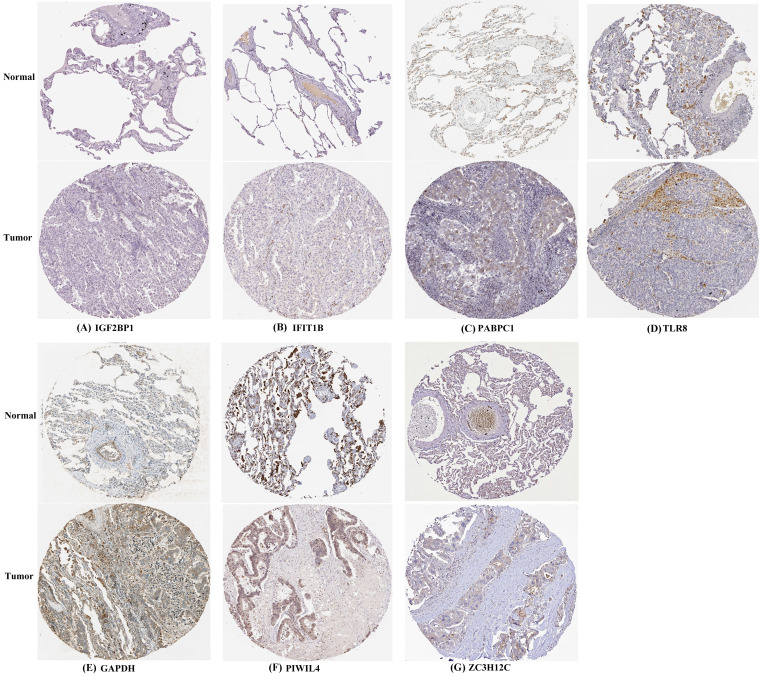
**Verification of hub RBPs expression in LUAD and normal lung tissue using the HPA database.** (**A**) IGF2BP1, (**B**) IFIT1B, (**C**) PABPC1, (**D**) TLR8, (**E**) GAPDH, (**F**) PIWIL4, (**G**) ZC3H12C.

